# Systematic review and meta-analysis of propofol versus barbiturates for controlling refractory status epilepticus

**DOI:** 10.1186/s12883-019-1281-y

**Published:** 2019-04-06

**Authors:** Qing Zhang, Yun Yu, Yu Lu, Hongli Yue

**Affiliations:** Department of Anesthesiology, Beijing Tiantan Hospital, Capital Medical University, No.119, Nansihuanxi Road, Fengtai District, Beijing, 100070 China

**Keywords:** Propofol, Barbiturates, Refractory status epilepticus, RSE, Meta-analysis

## Abstract

**Background:**

Several studies have compared the efficacy and safety of propofol and barbiturates in the treatment of refractory status epilepticus (RSE). This study aims to quantitatively assess the advantages and disadvantages of propofol and barbiturates in controlling RSE.

**Methods:**

We searched for studies with relevant data from the PubMed, Embase, Ovid, Cochrane Library, Springer Link, Web of Science, and China National Knowledge Infrastructure databases. By calculating odds ratios and standardized mean differences with 95% confidence intervals, we assessed the disease control rate (DCR), case fatality rate (CFR), average control time (ACT), average tracheal intubation placement time (ATIPT), and incidence of hypotension between propofol and barbiturates in treating RSE.

**Results:**

Seven studies with 261 patients were included in this analysis. Meta-analysis revealed that the DCR of propofol was higher than that of barbiturates (*p* < 0.001) and that the CFR (*p* = 0.382) between the two treatment did not significantly differ in controlling RSE. Propofol shortened the ACT (p < 0.001) of RSE and reduced the ATIPT (p < 0.001) of patients with RSE more extensively than did barbiturates and did not increase the incidence of hypotension (*p* = 0.737).

**Conclusions:**

In comparison with barbiturates, propofol can control RSE and shorten ATIPT in a more efficient and timely manner. Moreover, the drug does not increase the incidence of hypotension and CFR.

## Background

Refractory status epilepticus (RSE) is an acute critical illness characterized by recurrent or persistent epilepsy; it is a complex and rapidly progressing disease with a high mortality rate of 15% for RSE and 40% for super-RSE [1]. Clinically, RSE is defined as status epilepticus that continues or recurs 24 h or more after the onset of anesthetic therapy despite the administration of two appropriately selected and dosed antiepileptic drugs, including benzodiazepine [2–4]. Its most prominent feature is that it is insensitive to conventional antiepileptic drugs. First-line drugs for RSE treatment are currently being developed. When the preferred method is ineffective, multiple treatment methods are recommended for guiding follow-up treatment [5]. A number of intravenous anesthetic drugs are often used to treat RSE, such as barbiturates (e.g., pentobarbital, thiopental, etc.), midazolam, and propofol [5].

Due to its pharmacokinetic characteristics of rapid onset and short half-life, propofol is now used in the treatment of RSE [5]. However, propofol may also exert adverse reactions, such as hypotension, severe metabolic acidosis, and rhabdomyolysis; moreover, its safety has been questioned by some clinicians [6]. Many medical centers in Europe and the United States have used propofol to treat RSE and found increased risks of developing propofol infusion syndrome; reducing the amount of propofol by adding midazolam may help reduce the incidence of this syndrome [7]. Therefore, benzodiazepines and barbiturates appear to be the mainstream drugs for treating RSE [8]. At present, evidence-based medicinal data for the treatment of RSE with propofol are lacking. Some studies have compared the efficacy of propofol and barbiturates in the treatment of RSE, but the results have not been comparable [5–7]. Herein, we collected observational investigations to compare the efficacy of propofol and barbiturates for treating RSE and performed a systematic review and meta-analysis to quantitatively assess the advantages and disadvantages of these two treatments.

## Methods

### Literature search

We searched the following scientific medical databases for studies comparing the efficacy of propofol and barbiturates in the treatment of RSE: PubMed, Embase, Ovid, Cochrance Library, Springer Link, Web of Science, and China National Knowledge Infrastructure (CNKI). The time frame for the document search was from the date of database establishment to March 2018. The keywords used for retrieval included: “epilepsy,” “antiepileptic drugs,” “propofol,” “2,6-bisisopropylphenol,” “diisopropyl phenol,” “diprivan,” “disoprofol,” “barbiturates,” “phenobarbitol,” “thiopental,” “secobarbital,” “fosphenytoin,” “refractory status epilepticus,” “status epilepticus,” “generalized status epilepticus,” “disoprofol,” and “RSE.” The search strategy involved the combination of the topic of propofol with that of barbiturates and their combination with RSE. We applied Boolean operators, wildcards, and field identifiers to group search terms. Meta-analysis was then conducted according to the Preferred Reporting Items for Systematic Reviews and Meta-Analyses statement [9].

### Inclusion criteria

The inclusion criteria were as follows: (1) The patients under study must meet the diagnostic criteria for RSE of the International League Against Epilepsy [3]; (2) the study must be a controlled trial or at least a case-control study; (3) patient inclusion must not be limited by age and gender; (4) the study must clearly compare the efficacy of propofol and barbiturates for controlling RSE; and (5) the study should have at least one observation of (a) disease control rate (DCR), which is the rate of seizure control defined as a period of over 12 h in which clinical epileptic seizure or EEG seizure is terminated after medication, (b) case fatality rate (CFR), (c) average control time (ACT) and hypotension incidence, or (d) average tracheal intubation placement time (ATIPT) and provide data on means and standard deviations.

### Exclusion criteria

The exclusion criteria were as follows: (1) The study included patients with head injuries; (2) the sample size of the propofol and barbiturate groups was unclear; (3) research costs were provided by drug manufacturers or studies were initiated by drug producers; (4) the dose and time of administration of propofol and barbiturates were not indicated; (5) general treatment methods (rehydration, cooling, dehydration, reducing cranial pressure, maintenance of vital signs, and other routine treatment) for the two groups were not explained clearly or unbalanced, and (6) no clear observable data for efficacy evaluation were provided.

### Extraction of variables

The extracted data included: (1) Researchers, publication date, and country; (2) group of patients and the corresponding number of cases; (3) general demographics, such as age and gender; (4) Acute Physiology and Chronic Health Evaluation II (APACHE II); (5) the design of the study (retrospective or prospective) and the usage method and dose of propofol and barbiturates; and (6) the efficacy index of therapy (i.e., DCR, CFR, ACT, hypotension incidence, and ATIPT).

### Methodological assessment

We mainly adopted the criteria of the Cochrane Handbook (Version 5.0.1) to assess the quality of the included studies, which was special for the systematic review of interventions [10]. These criteria present a judgement and support for the judgement for each entry in a “risk of bias” table, where each entry addresses a specific feature of the study. The judgement for each entry involved assessment of the risk of bias as “low risk,” “high risk,” or “unclear risk,” with the last category indicating either a lack of information or uncertainty over the potential for bias [10]. Two investigators independently reviewed and determined the quality of each study. Discrepancies were resolved by consensus with a third expert.

### Statistical analysis

(1) We used the χ^2^ test and the I^2^ statistical test to determine whether the included literature had heterogeneity. When the results of the χ^2^ test indicated a *p* value greater than 0.10 and the I^2^ value was ≤50%, which indicates that the possibility of heterogeneity among studies is small, we chose the fixed effect model of meta-analysis; otherwise, we attempted to identify the cause of heterogeneity and used the random-effect model for meta-analysis. (2) For measurement data, we used standard mean differences (SMDs) and 95% confidence intervals (CIs) to estimate statistical effects; for count data, we use odds ratios (ORs) and 95% CIs. (3) The total effect of the meta-analysis was expressed using the *Z* value, which is considered statistically significant when *p* < 0.05. (4) We deleted studies one by one and recalculated the statistical effect of this deletion to determine whether a single study produced an impact on the overall statistical results. (5) We drew a funnel plot and performed Begg and Egger tests to determine the possibility of publication bias. (6) Meta-analysis was performed using Revman 5.2 (Cochrane Collaboration) and Stata 14.0 (Stata Corporation, TX, USA) software. General measurement statistics were completed using SPSS (version 21.0, Chicago, USA) software.

## Results

### Seven studies were qualified for this meta-analysis

**Fig. 1:**
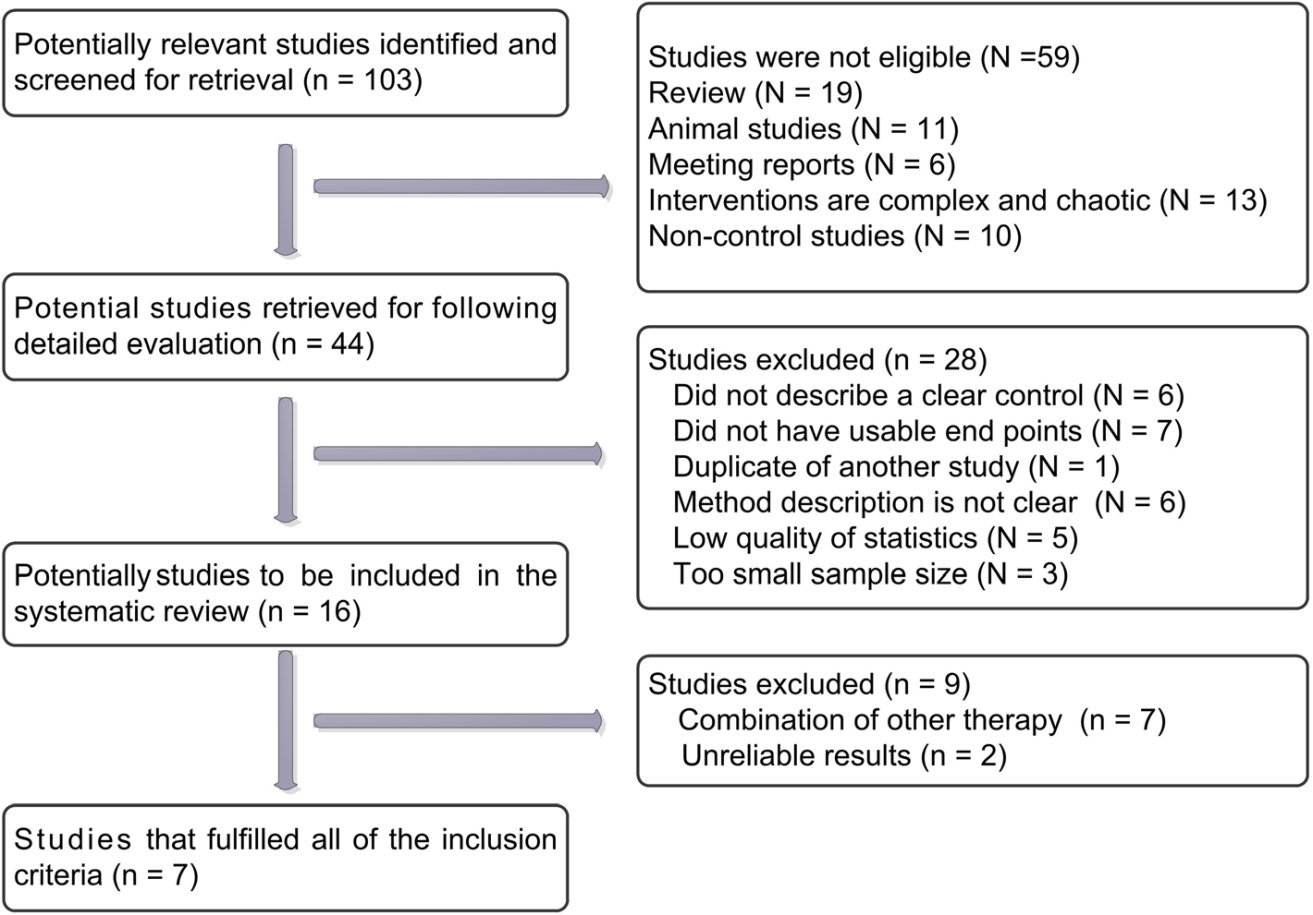
Flow chart of selection process for studies included in meta-analysis. At first, 103 investigations were defined to be closely correlated to the conception of this study. After a careful screening, a total of 7 studies met the inclusion criteria, which were searched from the database of Medline/PubMed, EMBASE, Cochrance Library, Web of Science and China National Knowledge Infrastructure Database

A total of 103 articles were considered closely correlated to the concept of this study. Based on the inclusion criteria, 59 articles, including reviews, animal studies, summary of meetings, and single-arm studies, were considered ineligible for the present study. Of the remaining 44 studies, another 28 documents were excluded because of the following reasons: lack of a clear control group (6 studies), lack of usable end points (7 studies), duplication of another study (1 study), unclear methodology (6 studies), unreliable statistical design (5 studies), and small sample size (3 studies). Hence, 16 studies were considered to meet our inclusion criteria. However, another 9 documents, including articles describing the simultaneous use of benzodiazepines (7 studies) and those lacking the observation data required for meta-analysis (2 studies), were eventually excluded from our corpus. Finally, 7 qualified studies [11–17] were subjected to meta-analysis (Fig. 1).

### The included studies presented good homogeneity and comparability in terms of demographics

As shown in Table 1, the seven included studies [11–17] were reported between 2005 and 2017 and involved a total of 261 patients. A total of 134 patients were enrolled in the propofol group, and 127 patients were enrolled in the barbiturate group. In the propofol group, 51.8% of the patients were male, while 48.2% were female. In the barbiturate group, 58.9% of the patients were male, while 41.1% were female. In addition, three studies [11–13] reported *APACHE* II data. Statistical analyses revealed that the demographics between the propofol and barbiturate groups did not significantly differ (*p* > 0.05).

**Table 1:** Description of the included studies

Authors	Year	Cases (N)			Age (Years)		Gender (Male/female) (N)				*APACHE* II	
		All	Propofol	Barbiturates	Propofol	Barbiturates	Propofol		Barbiturates		Propofol	Barbiturates
van Gestel JP [14]	2005	42	22	20	–	–	–	–	–	–	–	–
Xiuquan F [12]	2005	18	9	9	45.2	46.3	4	5	6	3	26.5	23.9
Zengming W [11]	2011	14	7	7	41.2	43.3	3	4	5	2	23.5	22.9
Rossetti AO [15]	2011	23	14	9	57 (26–87)	64 (16–78)	7	7	3	6	–	–
Xiang C [13]	2016	18	9	9	45.2 ± 2.35	45.0 ± 2.39	6	3	7	2	24.1	24
Chunfen Z [17]	2017	90	45	45	26.3 ± 2.5	26.6 ± 2.6	23	22	24	21	–	–
Linzhi C [16]	2017	56	28	28	40.86 ± 2.89	41.35 ± 3.15	15	13	18	10	–	–

N, cases, *APACHE* II, Acute Physiology and Chronic Health Evaluation II

### Methodological assessment indicated that the included studies had moderate to high study quality

**Fig. 2:**
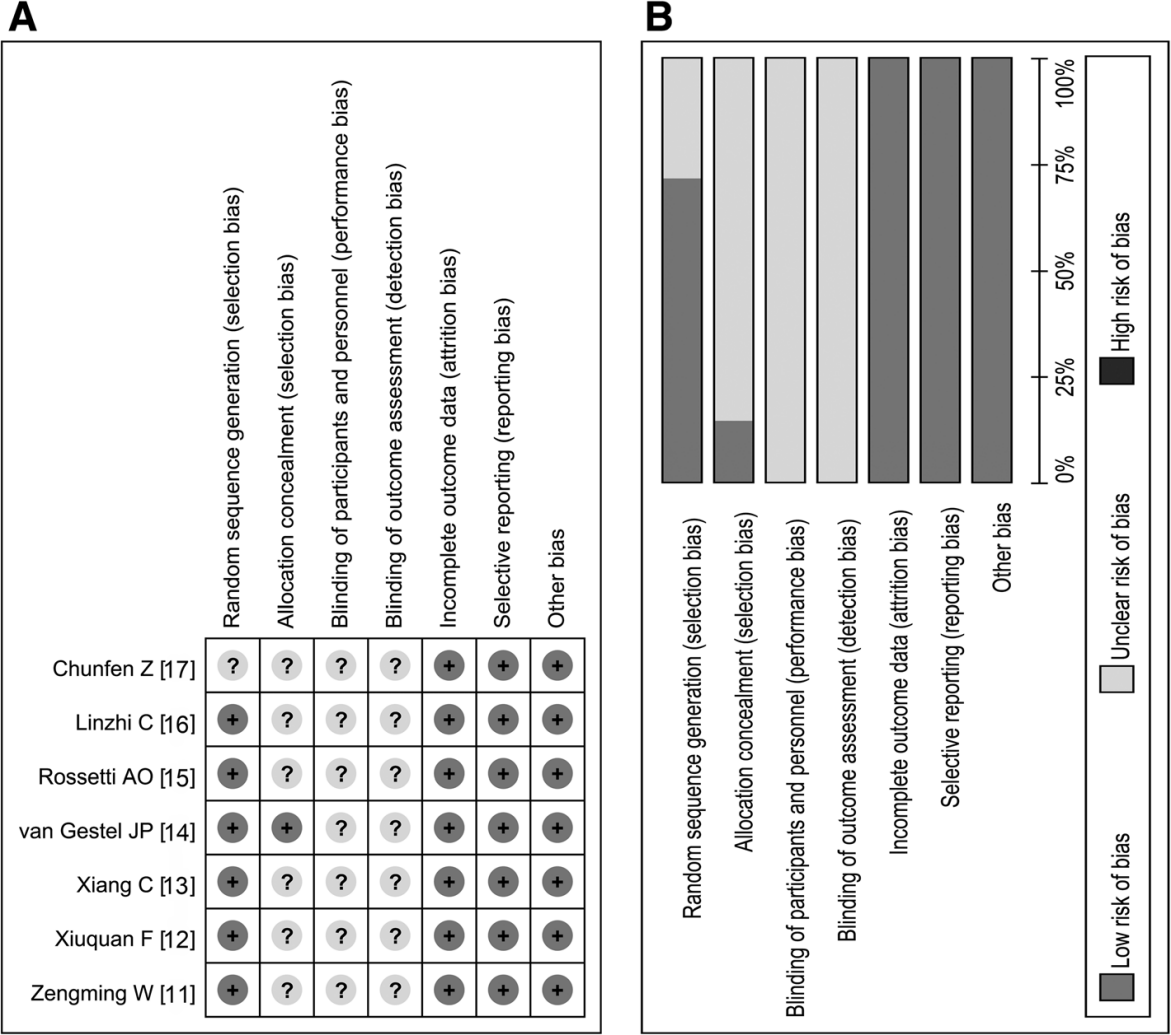
Quality assessment of included studies. **a** According to the Cochrane Handbook of Systematic Review, Summary analysis indicated that included studies had moderate to high study quality. **b** Except the blinding and allocation concealment, all of seven studies included did a good job in other areas and had moderate to high study quality

Table 2 shows that the seven included studies [11–17] were all retrospective in nature. Five studies [11–13, 16, 17] were reported in China, one was performed in the Netherlands [14], and another study originated from Switzerland [15]. Except for one study [15], the rest of the studies [11–14, 16, 17] provided detailed drug use, time, dose, and delivery pathways (Table 2). Nearly all of the included studies [11–17] provided key indicators for evaluating efficacy but did not reveal whether blinding and hidden allocation were performed. Summary analysis indicated that the included studies had moderate to high study quality (Fig. 2a and b).

**Table 2:** Methodology and quality of inclined studies

Authors	Year	Research design	Country	Interventions		Drug Management		Efficacy judgment
				Propofol	Barbiturates	Propofol	Barbiturates	
van Gestel JP [14]	2005	Retrospective	Netherlands	Propofol	Thiopental	First: 1–2 mg /kg IV; then 1–5 mg/(kg/h) maintenance	–	DCR, CFR
Xiuquan F [12]	2005	Retrospective	China	Propofol	Thiopental	First: 100 mg IV; then 6–12 mg/(kg/h) maintenance	First: 100 mg IV; then 3–5 mg/(kg/h) maintenance	DCR, ACT, hypotension, ATIPT, CFR
Zengming W [11]	2011	Retrospective	China	Propofol	Thiopental	First: 100 mg IV; then 6–12 mg/(kg/h), maintenance	First: 100 mg IV; then 3–5 mg/(kg/h) maintenance	DCR, ACT, ATIPT, CFR
Rossetti AO [15]	2011	Retrospective	Switzerland	Propofol	Barbiturates	–	–	DCR, ACT, CFR
Xiang C [13]	2016	Retrospective	China	Propofol	Thiopental	First: 100 mg IV; then 6–12 mg/(kg/h) maintenance	First: 100 mg IV; then 3–5 mg/(kg/h) maintenance	DCR, ACT, ATIPT, CFR
Chunfen Z [17]	2017	Retrospective	China	Propofol	Thiopental	First: 1–2 mg /kg IV; then 5-10 mg/(kg/h), maintenance	–	DCR, ACT, ATIPT,
Linzhi C [16]	2017	Retrospective	China	Propofol	Thiopental	First: 100 mg IV; then 6–12 mg/(kg/h) maintenance	First: 100 mg IV; then 3–5 mg/(kg/h) maintenance	DCR, ACT, hypotension, CFR

DCR, disease control rate; CFR, case fatality rate; ACT, average control time; ATIPT, average tracheal intubation placement time; kg, kilogram; h, hour; First, first dose

### Heterogeneity analysis

When comparing DCRs between propofol and barbiturates, the results of heterogeneity testing suggested a χ^2^statistic of 4.85 (degrees of freedom = 6; *p* = 0.563) and I^2^ statistic (due to the heterogeneity in OR changes) of 0%. When comparing CFRs, the results suggested a χ^2^statistic of 2.55 (degrees of freedom = 5; *p* = 0.768) and I^2^ statistic of 0%. These results indicate the absence of heterogeneity between groups. Hence, we used the fixed-effect model of meta-analysis with the data to calculate for overall effects. When comparing ACT and ATIPT, the results suggested χ^2^ statistics of 62.53 and 81.93 (both *p* < 0.001) and I^2^ statistics of 93.6 and 95.1%, respectively. Although these values indicate heterogeneity in the data, the included studies had very good homogeneity from the clinical design perspective, and their design and implementation were well comparable. Hence, we used the random-effects model of meta-analysis with the data to complete our analysis.

### The DCR of propofol was significantly higher than that of barbiturates in controlling RSE

**Fig. 3:**
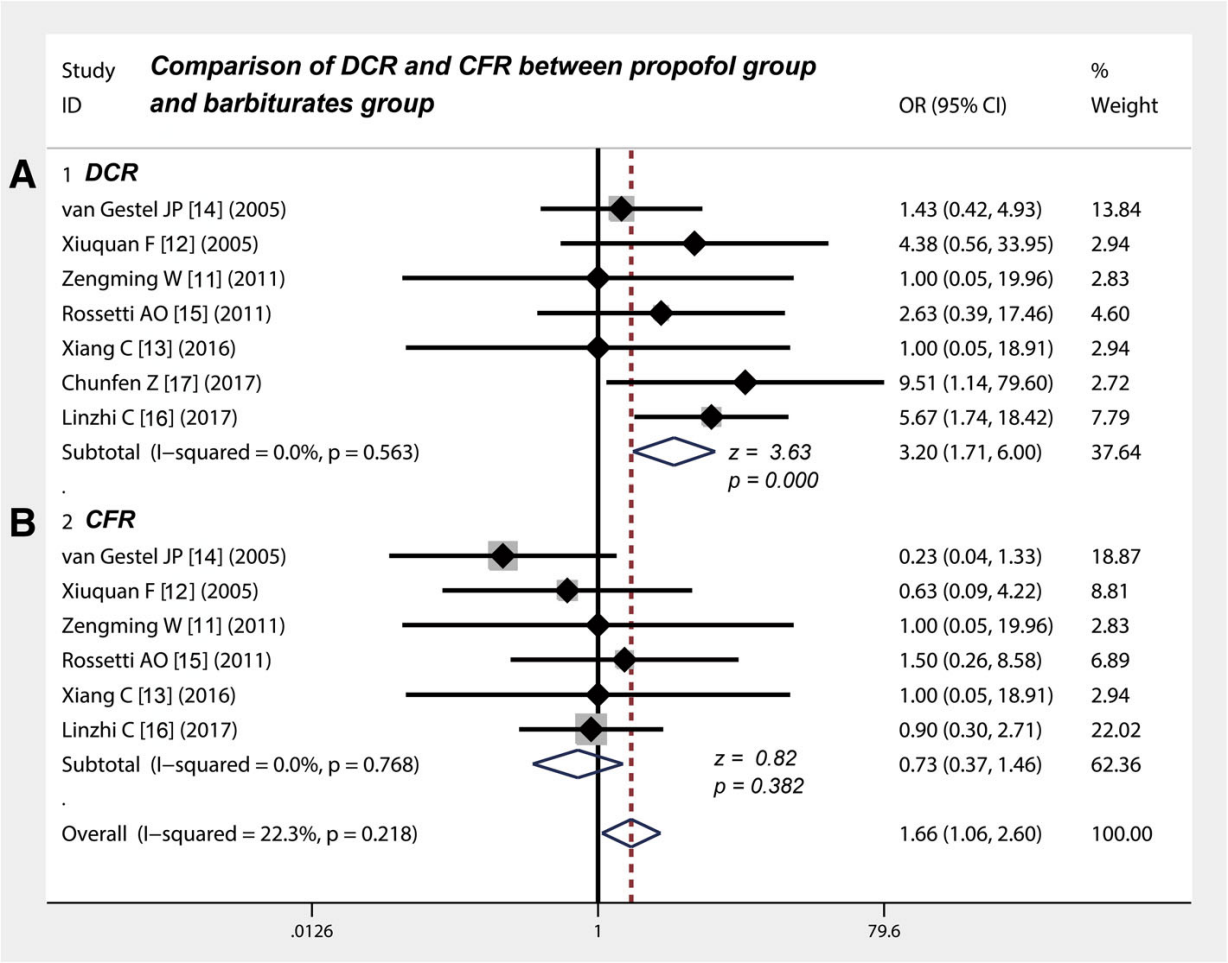
Comparison of DCR and CFR between propofol group and barbiturates group. **a** DCR of propofol is significantly higher than that of barbiturates in controlling RSE (z = 3.63, *P* < 0.001). **b** There is no significant difference in CFR between propofol and barbiturates in the treatment of RSE (z = 0.82, *P* = 0.382). CI, confidence interval; OR, odds ratio; DCR, disease control rate; CFR, case fatality rate

Table 3 shows that seven studies [11–17] compared DCR between propofol and barbiturate in treating RSE. The ORs of the included studies ranged from 1.0 to 9.51%, the pooled OR derived from the fixed-effect model of the meta-analysis was 3.20, and the 95% CIs were in the range of 1.71–6 (Fig. 3a). This finding indicates that the DCR of propofol is significantly higher than that of barbiturates in controlling RSE (*z* = 3.63, *p* < 0.001).

**Table 3:** The efficacy evaluation for propofol and barbiturates in controlling refractory status epilepticus

Author	Efficacy determination												Adverse reactions			
	DCR				ACT (minutes)		ATIPT (days)		CFR				Hypotension incidence			
	Propofol		Barbiturates		Propofol	Barbiturates	Propofol	Barbiturates	Propofol		Barbiturates		Propofol		Barbiturates	
	n/N	%	n/N	%					n/N	%	n/N	%	n/N	%	n/N	%
van Gestel JP [14]	14/22	64	11/20	55	–	–	–	–	2/22	9	8/20	40	–	–	–	–
Xiuquan F [12]	7/9	77.7	4/9	44.4	3.71 ± 1.1	116 ± 31	2 ± 0.08	6 ± 0.31	5/9	66.6	6/9	55.5	7/9	77.7	6/9	66.6
Zengming W [11]	6/7	85.7	6/7	85.7	5.62 ± 2.1	52 ± 17	3 ± 0.12	6 ± 0.27	1/7	14.3	1/7	14.4	–	–	–	–
Rossetti AO [15]	6/14	43	2/9	22	–	–	4	13.5	6/14	43	3/9	33	7/14	50	5/9	55
Xiang C [13]	8/9	88.8	8/9	88.8	5.58 ± 2.51	51.91 ± 2.49	3.01 ± 0.11	6.03 ± 0.19	1/9	11.1	1/9	11.1	–	–	–	–
Chunfen Z [17]	44/45	97.7	37/45	82.2	15.51 ± 1.41	24.39 ± 2.77	2.62 ± 1.21	3.61 ± 2.59	–	–	–	–	–	–	–	–
Linzhi C [16]	22/28	78.57	11/28	39.28	3.78 ± 1.1	52.6 ± 17.94	–	–	18/28	64.28	19/28	67.85	15/28	53.57	17/28	60.71

DCR, disease control rate; ACT, Average control time; ATIPT, Average tracheal intubation placement time; CFR, case fatality rate

### The CFR between propofol and barbiturates in the treatment of RSE did not significantly differ

Table 3 shows that six studies [11–16] compared the CFR between propofol and barbiturates in the treatment of RSE. The ORs of the included studies ranged from 0.23 to 1.5%, the pooled OR derived from the fixed effect model of meta-analysis was 0.73, and the 95% CIs were in the range of 0.37–1.46 (Fig. 3b). This finding indicates that the CFR between propofol and barbiturates in the treatment of RSE does not significantly differ (*z* = 0.82, *p* = 0.382).

### Propofol significantly shortened the ACT of RSE compared with barbiturates

**Fig. 4:**
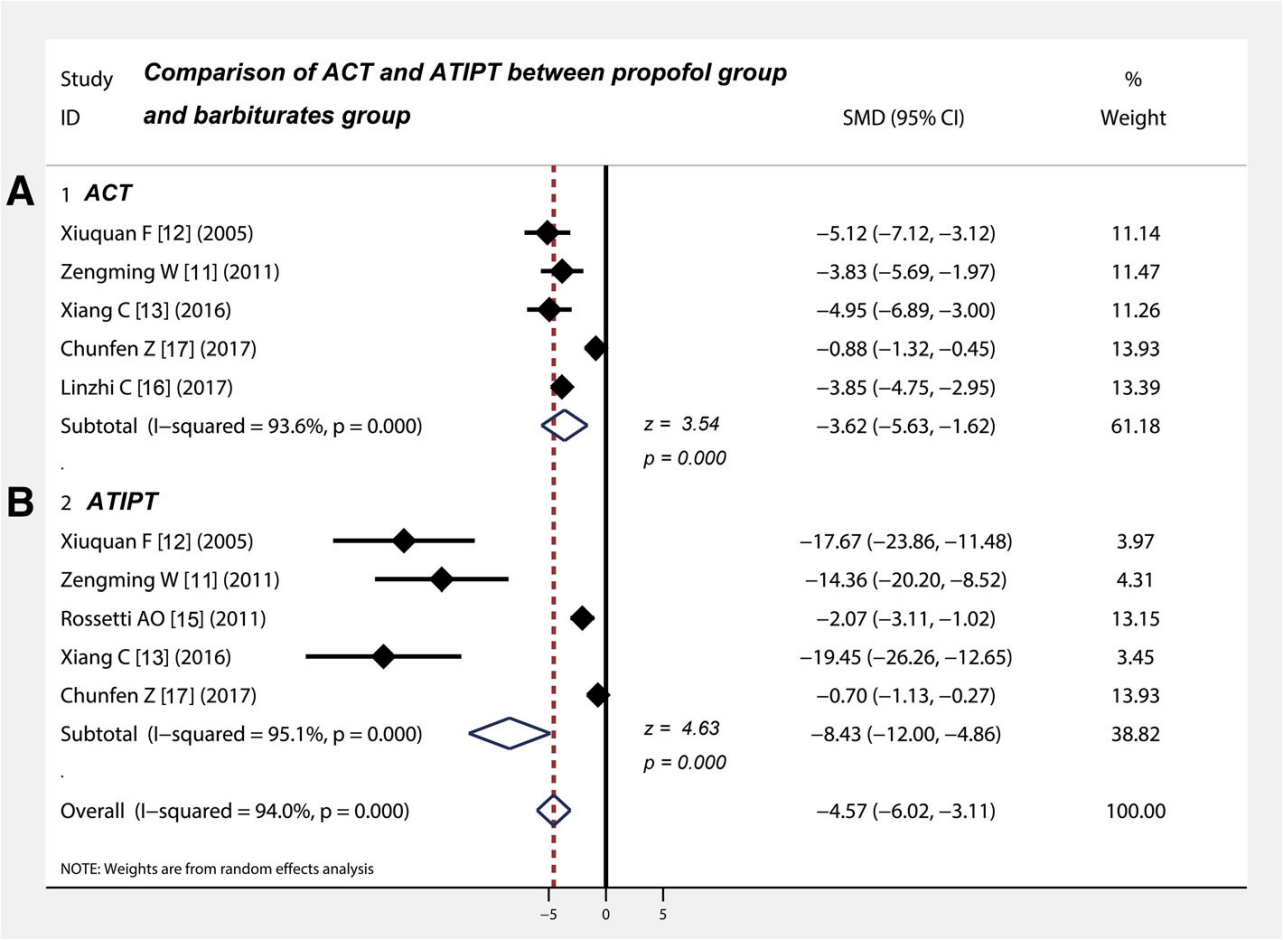
Comparison of ACT and ATIPT between propofol group and barbiturates group. **a** The ACT of the propofol group was significantly lower than that of the barbiturates group (z = 3.54, P < 0.001). **b** The ATIPT of the barbiturates group was significantly longer than that of the propofol group (z = 4.63, P < 0.001). CI, confidence interval; SMD, standard mean difference; ACT, average control time; ATIPT, average tracheal intubation placement time

Table 3 shows that five studies [11–13, 16, 17] compared the ACT of RSE between propofol and barbiturates in the treatment of RSE. The random-effect combined SMD was − 3.62 and the 95% CIs were in the range of − 5.63 to − 1.62, which shows that the ACT of propofol is significantly less than that of the barbiturates (*z* = 3.54, *p* < 0.001) (Fig. 4a). These results indicate that propofol significantly shortens the ACT of RSE compared with barbiturates.

### Propofol obviously reduced the ATIPT of patients with RSE compared with barbiturates

Table 3 shows that five studies [11–13, 15, 17] compared the ATIPT of patients with RSE between propofol and barbiturates. The random-effect combined SMD was − 8.43 (95% CI: − 12 to − 4.86; *z* = 4.63, *p* < 0.001), which means the ATIPT of the barbiturate group is significantly longer than that of the propofol group (z = 4.63, *p* < 0.001) (Fig. 4b).

### Propofol did not increase the incidence of hypotension compared with barbiturates

**Fig. 5:**
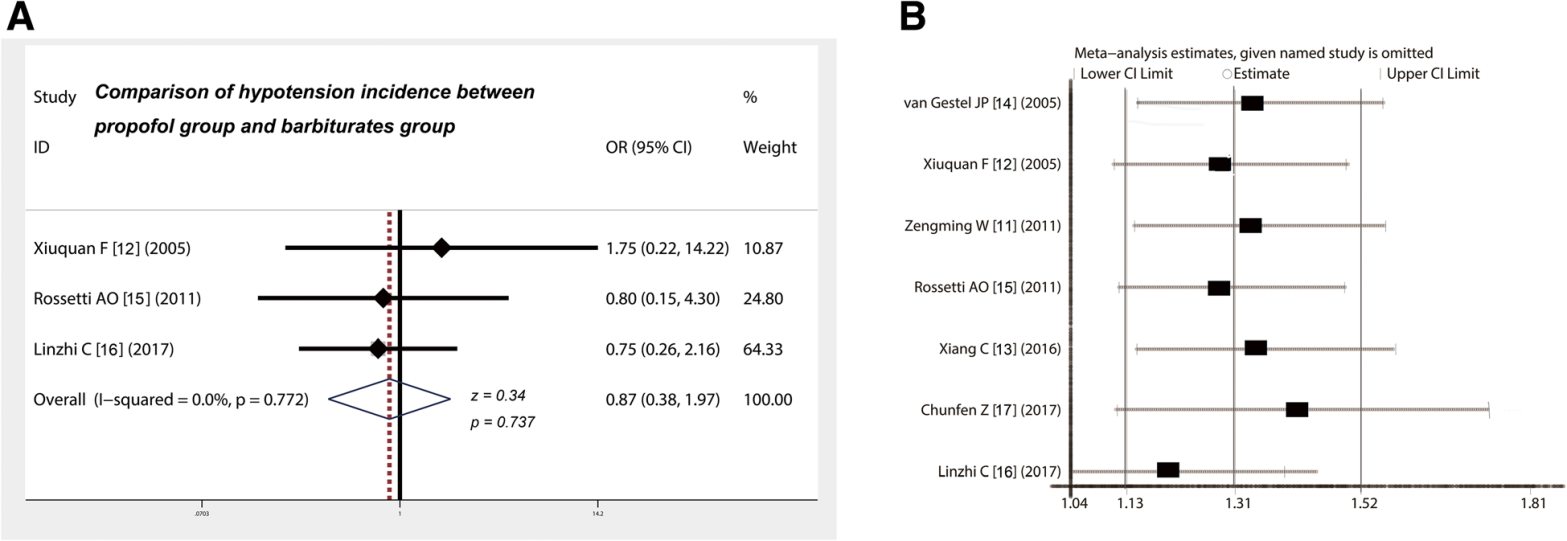
Comparison of hypotension incidence between propofol group and barbiturates group and sensitivity analysis of included studies. **a** There was no significant difference in the incidence of hypotension between propofol and barbiturates in the treatment of RSE (z = 0.34, *P* = 0.737). **b** Exclusion of individual studies did not substantially alter the estimates and affect the final statistical performance. CI, confidence interval; OR, odds ratio

Table 3 shows that three studies [12, 15, 16] compared the incidence of hypotension between propofol and barbiturates in the treatment of RSE. The ORs of the included studies ranged from 0.75 to 1.75%, the pooled OR derived from the fixed-effect model of the meta-analysis was 0.87, and the 95% CIs were in the range of 0.38–1.97 (Fig. 5a). This finding indicates that the incidence of hypotension between propofol and barbiturates does not significantly differ in the treatment of RSE (*z* = 0.34, *p* = 0.737).

### Analysis of sensitivity

To determine whether individual studies included directly affected the overall statistical effect, we conducted a series of sensitivity analyses on these indicators. Table 4 shows that the largest estimate was 1.42, whereas the lowest estimate was 1.2. The estimates for each study were basically distributed on both sides of the average estimate (1.31). The results suggest that exclusion of individual studies does not substantially alter estimates or affect the final statistical performance.

**Table 4:** Sensitivity analysis of included studies

Study omitted	Year	Estimate	95% confidence interval	
			Lower limit	Upper limit
van Gestel JP [14]	2005	1.3398983	1.1499814	1.5611796
Xiuquan F [12]	2005	1.2905582	1.1106998	1.4995416
Zengming W [11]	2011	1.3389788	1.1449463	1.5658935
Rossetti AO [15]	2011	1.2942261	1.1188998	1.4970251
Xiang C [13]	2016	1.3484002	1.1487684	1.5827239
Chunfen Z [17]	2017	1.4206468	1.1158725	1.808663
Linzhi C [16]	2017	1.2040412	1.0382174	1.3963504
Combined		1.3135422	1.1318713	1.5243721

### Analysis of publication bias

**Fig. 6:**
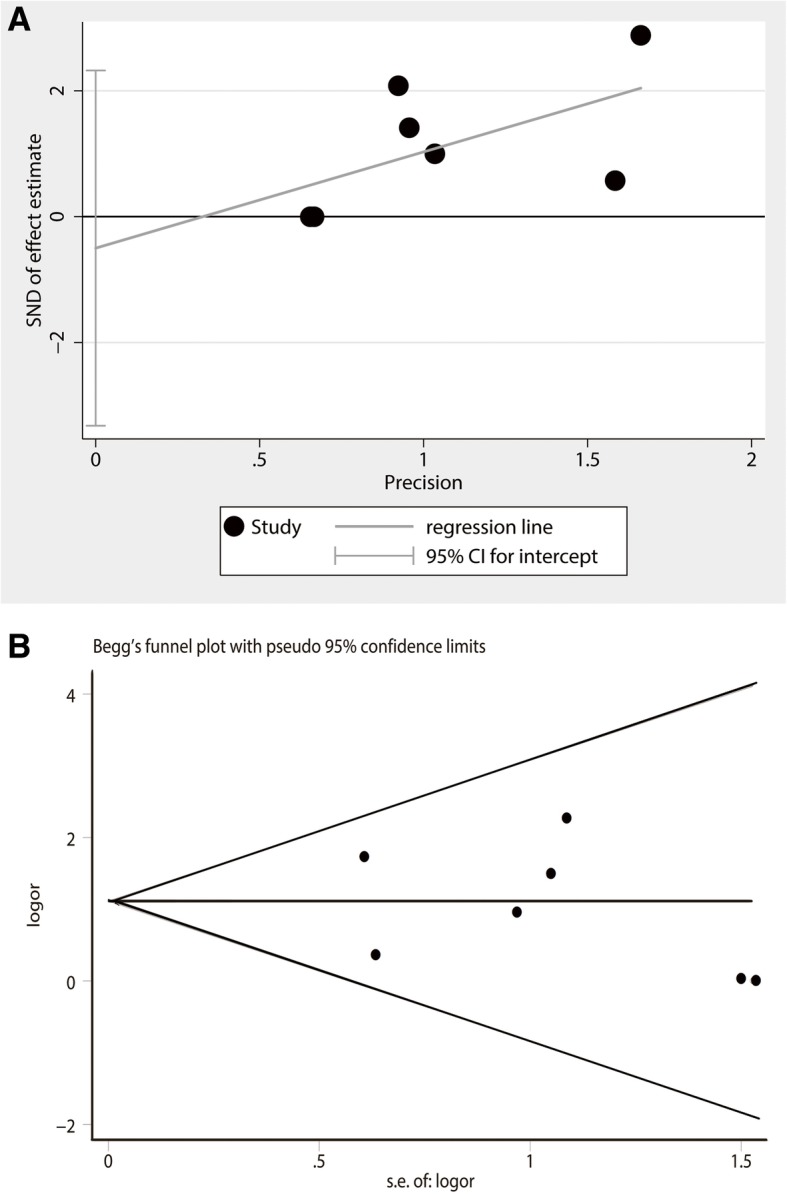
Publication bias analysis on included studies. **a** The distribution graph derived from Egger test showed that included studies were basically distributed on both sides of the baseline. **b** The funnel plot derived from begg test indicated that all 7 studies were symmetrically distributed on both sides of the funnel plot. CI, confidence interval

The results of Egger testing suggested a *T* value of − 0.46 (Pr > |*t*| = 0.668), while the results of Begg testing demonstrated that Pr > |*z*| = 0.652 with a standard deviation of 6.66 (Pr > |*z*| = 0.764). The distribution graph derived from the Egger test showed that the included studies were evenly distributed on both sides of the baseline (Fig. 6a). In addition, the funnel plot derived from the Begg test indicated that all seven studies were symmetrically distributed on both sides of the funnel plot (Fig. 6b). Hence, the studies included in the present meta-analysis do not have publication bias.

## Discussion

In recent years, the reactivity of anti-epileptic drug treatments has gradually attracted researchers’ attention in the treatment of status epilepticus. Approximately 23–48% of all patients with status epilepticus may experience seizures after standard first- and second-line treatment [2, 18]. Patients in epileptic states with over 2 h of continuous epileptic seizures beyond the control of first- and second-line antiepileptic drugs may need to be administered anesthetic drugs for treatment [19]. Propofol presents the advantage of high lipophilicity; it has a large volume of distribution, and it can be rapidly taken up and eliminated in the central nervous system. These characteristics confer the drug a certain advantage in the treatment of RSE [7, 20]. Some studies have specifically investigated the clinical effect of propofol versus barbiturates in treating RSE. In the present work, we performed a systematic literature review and meta-analysis to assess the clinical benefit of propofol in controlling RSE.

Seven studies were included in this meta-analysis according to the established inclusion and exclusion criteria; in total, these studies reported 134 patients in the propofol group and 127 in the barbiturate group. Statistical analysis of the demographics between the propofol and barbiturate group indicated that the included studies were of good homogeneity and comparability. Except for blinding and allocation concealment, which may be mainly due to medical ethics restrictions and the severity of RSE, all seven studies were effective in other areas and methodology. In the heterogeneity analysis of count data (DCR, CFR, and hypotension incidence), no statistical heterogeneity was found between studies. However, two indicators of measurement data (ACT and ATIPT) revealed statistical heterogeneity. Hence, the heterogeneity observed may have originated from differences in drug dose and disease severity. Overall, however, the data obtained showed very good homogeneity from the clinical design perspective, and the design and implementation of these studies were well comparable. Our sensitivity analysis indicated that exclusion of individual studies does not substantially alter estimates or affect the final statistical performance. Egger and Begg test results also proved that the studies included in the present meta-analysis did not have publication bias.

Our study showed that the DCR (DCR is the rate of seizure control, defined as a period of more than 12 h in which clinical epileptic seizure or EEG seizure are terminated after medication) of propofol is significantly higher than that of barbiturates in controlling RSE; CFR did not significantly differ between groups. Barbiturates increase the activity of inhibitory gamma-aminobutyric acid (GABA), hinder the effect of glutamate, inhibit the brain stem reticular structure activation system, and reduce the excitability of the cerebral cortex [21]. Thus, they could be used to treat RSE when other drugs fail to control it [22]. Propofol is a non-barbiturate intravenous anesthetic with high lipid solubility and a plasma protein binding rate of 97–98%. It has been proven to exert strong antiepileptic effects. The mechanism of action of this drug may include: (1) unique inhibition of the central nervous system, (2) enhanced GABA-mediated synaptic inhibition, and (3) reduced excitatory neurotransmitters (glutamate and aspartate) [23, 24]. A meta-analysis in 2002 showed that the efficacy, CFR, and residual function loss among patients with RSE treated with propofol, pentobarbital, and midazolam do not significantly differ [25]. We found that propofol could significantly increase the DCR of RSE and does not increase the CFR of patients compared with barbiturates, which indicates its advantage over barbiturates in controlling RSE.

Failure to control RSE in a timely manner often leads to disturbance of consciousness in patients with high fever, metabolic acidosis, hypoglycemia, shock, electrolyte imbalance, myoglobinuria, multiple organ failure, autonomic nerves disorder, and other life-threatening conditions [8, 13, 18, 25]. Therefore, terminating RSE as soon as possible is an important means to reduce the CFR and disability rate. Our study suggests that propofol significantly shortens the ACT of RSE compared with barbiturates, which means the former is more effective than the latter in controlling RSE. Propofol terminates both clinical and electric seizures quickly, but the maintenance of burst-suppression EEG patterns requires repetitive titration of doses [26]. In adult RSE therapy, propofol exerts control efficacy toward RSE similar to that of midazolam but is superior to barbital, can shorten recovery times, and accelerate the recovery of neuromotor and cognitive functions [27]. Endotracheal intubation is an extremely important aspect of RSE therapy. Our results indicated that propofol is superior to barbiturates in both indicators of ACT and ATIPT because it shortens the ACT of RSE and quickly terminates RSE, which would reduce the degree of damage to the patient’s brain and body. In addition, the ATIPT of patients with RSE in the propofol group was significantly shortened compared with that of patients in the barbiturate group, thereby indicating that patients in the former group recovered quickly and propofol reduces complications. The duration of mechanical ventilation in patients directly affects the occurrence of ventilator-associated pneumonia (VAP), and shortening the duration of mechanical ventilation is an important measure to reduce the incidence of VAP [28]. Prolonged ATIPT in the barbiturate group is well explained by its long half-life. Barbiturates act by enhancing the action of GABA-alpha receptor, which may exert a neuroprotective role and is beneficial to SRSE. The main disadvantage of barbiturates, however, is their rapid redistribution, which leads to accumulation and prolonged half-lives that can reach hours or days [29]. Furthermore, prolonged intubation is an established cost-driving factor in RSE. Hence, the use of propofol might lower the cost of RSE in the long run [30].

Although propofol features good pharmacokinetic characteristics, cardiovascular tolerance, and significant antiepileptic effects, it can also cause hypotension and propofol infusion syndrome [6, 7]. Previous studies have also revealed that the advantages of barbiturates in the treatment of RSE include their high efficiency and low relapse rate; some of its disadvantages, however, include a high incidence of hypotension and long mechanical ventilation [26, 31]. Our study showed that the incidence of hypotension did not significantly differ between propofol and barbiturates during treatment of RSE. Research has proposed that long-term infusion of propofol may induce propofol infusion syndrome, which is mainly manifested by severe metabolic acidosis, rhabdomyolysis, acute renal failure, refractory heart failure, and hyperlipidemia [32]. In our meta-analysis, none of the studies mentioned propofol infusion syndrome, although this finding may be due to reporting bias. Larger, randomized, controlled trials are needed to observe this side effect. Other adverse effects of propofol may include fever, transient dystonia, increased muscle enzymes, and increased blood lipids [6, 8, 24]. In our meta-analysis, except for hypotension, none of the included studies reported other side effects, which implies that the short-term use of propofol does not cause serious adverse events.

A recent study provided class III evidence that patients with SE receiving five IV anesthetic drugs (IVADs) have a higher proportion of infections and increased risk of death compared with patients not receiving IVADs [8]. Relevant guidelines indicate that, during treatment of convulsive RSE, continuous IVADs (CIVADs) should be considered to induce therapeutic coma (TC) when benzodiazepines and non-sedative antiepileptic drugs fail as initial treatment [33]. Induction of TC is applied to stop seizure activity and hypothetically prevent seizure-induced brain damage and reduce cerebral metabolism. However, one study from the United States and Europe on the use of TC for RSE treatment indicated that IVADs do not affect mortality but increase length of hospital stay [33]. At present, the level of evidence of the effectiveness and safety of narcotic drugs, such as propofol, barbiturates, and midazolam, remains relatively low in the treatment of RSE. Each anesthetic presents its own characteristics and risks. The specific anesthetic used in this research to control RSE is currently not supported by high-quality medical evidence.

Some deficiencies in the current study must be noted. First, most of the included studies contained relatively small sample sizes. Second, subtle differences in the use of drugs were noted between studies, and these differences may lead to methodological heterogeneity. Third, most of the studies included in our analyses originated from China, and the results obtained may include geographical bias. Fourth, except for hypotension, none of the studies provided specific data on other adverse effects of propofol and barbiturates. Finally, none of the studies included in this meta-analysis provided time data of the recurrence of epilepsy. Hence, future research should focus on these limitations.

## Conclusion

The DCR of propofol was higher than that of barbiturates, and the CFR between the two treatments did not significantly differ in controlling RSE. Compared with barbiturates, propofol shortened the ACT of RSE and reduced the ATIPT of patients with RSE more extensively, and the drug did not increase the incidence of hypotension. Open, multicenter, randomized controlled trials are necessary to provide more robust and powerful clinical evidence of the effects of profopol on RSE.

## Abbreviations

ACT: Average control time
ATIPT: Average tracheal intubation placement time
CFR: Case fatality rate
CI: Confidence interval
CNKI: China National Knowledge Infrastructure
DCR: Disease control rate
GABA: Gamma-aminobutyric acid
M ± SD: Mean ± standard deviation
OR: Odds ratio
RSE: Refractory status epilepticus
SMD: Standardized mean difference
